# Biogenic silver nanoparticles eradicate of *Pseudomonas aeruginosa* and Methicillin-resistant *Staphylococcus aureus* (MRSA) isolated from the sputum of COVID-19 patients

**DOI:** 10.3389/fmicb.2023.1142646

**Published:** 2023-04-06

**Authors:** Nahed M. Hawsawi, Arshad M. Hamad, Sahar N. Rashid, Fatma Alshehri, Mohamed Sharaf, Shadi A. Zakai, Sulaiman A. Al Yousef, Ahmed Mohamed Ali, Amira Abou-Elnour, Abdulsalam Alkhudhayri, Nadia Gouda Elrefaei, Amr Elkelish

**Affiliations:** ^1^Department of Clinical Laboratory Sciences, College of Applied Medical Sciences, Taif University, Taif, Saudi Arabia; ^2^Department of Biology, College of Science, Tikrit University, Tikrit, Iraq; ^3^Department of Physics, College of Science, Tikrit University, Tikrit, Iraq; ^4^Department of Biology, College of Science, Princess Nourah Bint Abdulrahman University, Riyadh, Saudi Arabia; ^5^Department of Biochemistry, Faculty of Agriculture, Al-Azhar University, Cairo, Egypt; ^6^Department of Biochemistry and Molecular Biology, College of Marine Life Sciences, Ocean University of China, Qingdao, China; ^7^Department of Clinical Microbiology and Immunology, Faculty of Medicine, King Abdulaziz University, Jeddah, Saudi Arabia; ^8^Department of Clinical Laboratory Sciences, College of Applied Medical Sciences, University of Hafr Al Batin, Hafar Al Batin, Saudi Arabia; ^9^Department of Biology, Deanship of Preparatory Year, Shaqra University, Shaqra, Saudi Arabia; ^10^Department of Botany and Microbiology, Faculty of Science, Zagazig University, Zagazig, Egypt; ^11^Department of Biology, College of Science, University of Hafr Al Batin, Hafar Al Batin, Saudi Arabia; ^12^Clinical Skills Laboratories, College of Medicine, Imam Abdulrahman Bin Faisal University, Dammam, Saudi Arabia; ^13^Department of Biology, College of Science, Imam Mohammad Ibn Saud Islamic University (IMSIU), Riyadh, Saudi Arabia; ^14^Department of Botany and Microbiology, Faculty of Science, Suez Canal University, Ismailia, Egypt

**Keywords:** bio-reducing agents, biosynthetic AgNPs, COVID-19, antibacterial activity, anticancer activity

## Abstract

In recent investigations, secondary bacterial infections were found to be strongly related to mortality in COVID-19 patients. In addition, *Pseudomonas aeruginosa* and Methicillin-resistant *Staphylococcus aureus* (MRSA) bacteria played an important role in the series of bacterial infections that accompany infection in COVID-19. The objective of the present study was to investigate the ability of biosynthesized silver nanoparticles from strawberries (*Fragaria ananassa* L.) leaf extract without a chemical catalyst to inhibit Gram-negative *P. aeruginosa* and Gram-positive *Staph aureus* isolated from COVID-19 patient’s sputum. A wide range of measurements was performed on the synthesized AgNPs, including UV–vis, SEM, TEM, EDX, DLS, ζ -potential, XRD, and FTIR. UV-Visible spectral showed the absorbance at the wavelength 398 nm with an increase in the color intensity of the mixture after 8 h passed at the time of preparation confirming the high stability of the FA-AgNPs in the dark at room temperature. SEM and TEM measurements confirmed AgNPs with size ranges of ∼40-∼50 nm, whereas the DLS study confirmed their average hydrodynamic size as ∼53 nm. Furthermore, Ag NPs. EDX analysis showed the presence of the following elements: oxygen (40.46%), and silver (59.54%). Biosynthesized FA-AgNPs (ζ = −17.5 ± 3.1 mV) showed concentration-dependent antimicrobial activity for 48 h in both pathogenic strains. MTT tests showed concentration-dependent and line-specific effects of FA-AgNPs on cancer MCF-7 and normal liver WRL-68 cell cultures. According to the results, synthetic FA-AgNPs obtained through an environmentally friendly biological process are inexpensive and may inhibit the growth of bacteria isolated from COVID-19 patients.

## 1. Introduction

Since the outbreak of COVID-19, it has posed a severe threat to global public health as approximately 200 million cases carried out by the virus causing 4 million deaths (Sposito et al., 2021). Secondary bacterial infection is an inevitable result of pulmonary dysbiosis and respiratory tract distortion caused by SARS-CoV-2 infection, making it a fatal disease (Hanada et al., 2018; Jose and Desai, 2020). Illustratively, SARS-CoV-2 infection creates a respiratory environment that permits the invasion of the abundance of bacterial pathogens through a distinct shift in the composition of the microbiome (Rhoades et al., 2021), with genetic traits suitable for chronic infections (Qu et al., 2022). This phase causes exacerbation of illness, and the clinical diagnosis and treatment are more complicated (Bosch et al., 2013), causing morbidity and mortality of critical patients to insanely raise (Sharifipour et al., 2020). It was also found that bacterial coinfection was more associated with patients being critically ill as well as staying a long time in the hospital environment (Abdoli et al., 2021).

*Pseudomonas aeruginosa* was notified as the most abundant superinfecting bacterial in the sputum samples and bronchoalveolar lavage fluids (BALF) (Qu et al., 2021; Rhoades et al., 2021). In this context, *Rothia* comes next in existence in the nasal microbiome, and it is considered the best predictor of SARS-CoV-2 vRNA infection in patient samples. Other species were also detected, including *Klebsiella pneuminiae* (Kaul et al., 2020), *Streptococcus pneumoniae* (Lansbury et al., 2020), *Staphylococcus aureus*, *Escherichia coli*, *Stenotrophomonas maltophilia* (Chong et al., 2021), *Mycoplasma pneumonia*, *Acinetobacter baumannii*, *Haemophilus influenzae* (Lansbury et al., 2020), and *methicillin-sensitive-Staphylococcus-aureus* (MRSA) (Yang et al., 2015). *Pseudomonas aeruginosa* is a Gram-negative bacterium (He et al., 2020). *P. aeruginosa* causes a very high rate of late-onset ventilator-associated pneumonia (VAP) in severe COVID-19 patients who require ECMO (Luyt et al., 2020). *P. aeruginosa* affects the patient through various mechanisms, thorough secretion of various virulence factors and forming a biofilm essential for host adaptation (Wang K. et al., 2017). These isolates have novel epigenetic markers and could form extravagant modifications that induce antibiotic resistance, persistent *in vivo* colonization, and disease induction in COVID-19 patients (Qu et al., 2021). These developments enable it to compete in polymicrobial environments and escape the host immune system attack (Gellatly and Hancock, 2013). Analysis of these evolutionary traits contributes to the prognosis of disease development and the required therapeutic measures to treat infections caused by *P. aeruginosa* co-infection in COVID-19 patients (Qu et al., 2022).

Methicillin-resistant *Staphylococcus aureus* (MRSA) is among the most frequent causative agents of pulmonary infection in COVID-19 patients (Bassetti et al., 2022), which causes multifocal pneumonia and right-sided empyema. *Staphylococcus aureus* also causes endocarditis, bacteremia, sepsis, and death, so it is considered a source of danger in the hospital environment for its risk of deadly outcomes in healthcare-associated infections (Punjabi et al., 2021). A wide diversity of patient- and environment-specific factors plays a role in the predominance of *S. aureus* coinfections in COVID-19 patients. It has been found that the COVID-19 treatment course is the major cause of this plethora as it includes intubation, mechanical ventilation, central venous catheter placement, and corticosteroids, which act as an excuse to introduce a foreign body or immunosuppressive properties that dually support bacterial growth (Monegro et al., 2021).

As *P. aeruginosa*, as we mentioned before, the aggregation of the environmental changes and frailty of the immune responses create suitable conditions for Staphylococcus aureus infection (Mulcahy and McLoughlin, 2016). Some strains of *Staphylococcus aureus* in COVID-19 patients secrete Panton-Valentine leukocidin (PVL), a cytotoxin that causes necrotizing pneumonia (Duployez et al., 2020). We conclude from the above that the risk of *P. aeruginosa* infection and high carriage of MRSA worsen the pathogenic condition and contribute to significantly raising the mortality rate of COVID-19 patients (He et al., 2020). Consequently, the search for bacterial pathogens coinfection treatments becomes the need of the hour.

**FIGURE 1:**
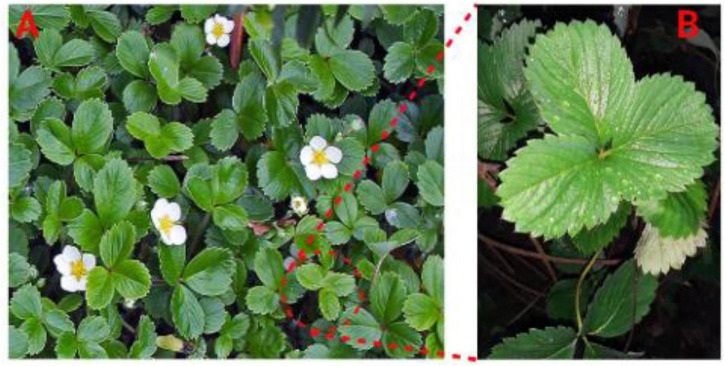
**(A)** Fragaria genus (*Rosaceae* family) strawberries (*Fragaria ananassa* L.) plant; **(B)** *Fragaria ananassa* L. leaf extract using to biosynthesized AgNPs.

Since ancient times plants have been essential bioactive sources. Thanks to their “phytochemicals.” Plants have a notable therapeutic value (Nishtha et al., 2017) and show healing power from diseases and disorders in a tremendously expansive range of health-related purposes (Punjabi et al., 2015). Approximately 12,000 such compounds have been isolated so for the Fragaria genus (*Rosaceae* family) (Sumaira et al., 2011), commonly known as strawberry (*Fragaria ananassa* L.) (Figures 1A, B), demonstrated as one of the most cosmopolitan important food plants with therapeutic potential all over the world (Liston et al., 2014). This plant has some characteristics that make it at the top of the dietary consumption rate, including taste, nutritional values, aroma (Awad and de Jager, 2003) and the high level of vitamin C, anthocyanins, ellagic acid, and other antioxidant compounds (Jakobek et al., 2007).

There are approximately 247 varieties known and listed in this genus. One of the most important species is *Fragaria x ananassa*. Duchesne, known as the garden strawberry, is native to northern America and cultivated all over the world (Liston et al., 2014). Previous studies have concluded that its leaves could be a good source for isolating active phytochemicals used in different medicinal approaches (Khan et al., 2018). Seventeen phenolic compounds were isolated from the methanolic extract of strawberry leaves and identified as hydrolysable tannins, flavanol, flavonol glycosides, and phenolic acids (El-Mesallamy et al., 2013).

Include these phenolic compounds in the diary diet as natural antioxidants protecting the human body from diverse diseases and infections like delayed senescence caused by oxidative stress, and other properties such as anticancer, anti-atherosclerotic, anti-neurodegenerative, anti-inflammatories, anti-tumors, antihistaminics, and antimicrobial activity (Seeram et al., 2006). Strawberry fruits exhibited antibacterial activity against various species such as *Micrococcus luteus, Escherichia coli, S. aureus, B.bronchiseptica, Kelbsiella sp.*, *Pseudomonas, Salmonella typhi*, and *P. aeruginosa* (Khan et al., 2018; Selim et al., 2022). In contrast, *Staphyllococcus aureus* is the highest resistance species to the plant extract. Moreover, the antimicrobial activities of the plant leave extracts were investigated against some bacterial strains, and the results showed inhibitory effects for most bacterial spp such as *Pseudomonas aeruginosa, Kelbsiella sp., Salmonella typhi, Staphyllococcus aureus, Escherichia coli* and *Enterococcus faecium* (VREfm) (El-Mesallamy et al., 2013; Alshawwa et al., 2022; Kandeel et al., 2022).

Nanoparticles are created with special qualities that make them useful in biology and materials research (Kesharwani et al., 2018; Sharaf et al., 2022b). Silver nanoparticles are among many that have attracted the greatest attention for research in recent years (Saravanan et al., 2018). Silver nanoparticles emit silver ions, which may destroy bacteria (Bapat et al., 2018). Due to electrostatic attraction and sulfur protein affinity, silver ions stick to the cell wall and cytoplasmic membrane. Adhered ions increase cytoplasmic membrane permeability and damage the bacterial envelope (Khorrami et al., 2018).

After free silver ions enter cells, respiratory enzymes may be inhibited, producing reactive oxygen species but interfering with adenosine triphosphate formation (Ramkumar et al., 2017). Reactive oxygen species may majorly cause cell membrane rupture and deoxyribonucleic acid (DNA) alteration. Because sulfur and phosphorus are key components of DNA, the interaction of silver ions with these elements may create issues with DNA replication, cell reproduction, and even the termination of microorganisms. Furthermore, silver ions may impede protein production by denaturing ribosomes in the cytoplasm (Darroudi et al., 2014).

**SCHEME 1:**
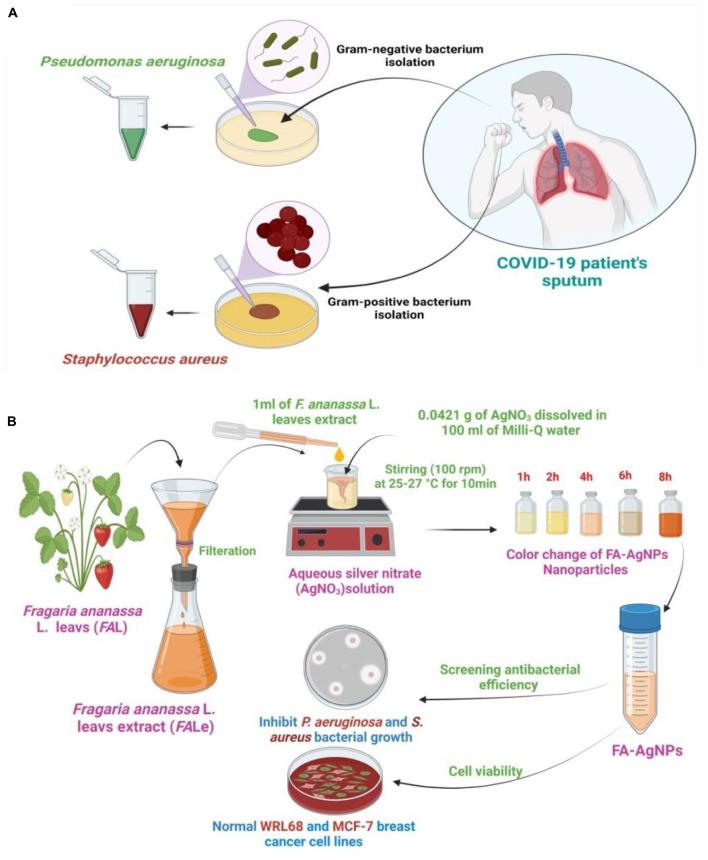
**(A)** Purification and isolation of pathogenic bacteria from COVID-19 patients and culture of the microorganism; **(B)** illustration of prepared FA–AgNPs biosynthesis at 25–27°C.

This work investigated the efficacy of biosynthesized silver nanoparticles from strawberries (*Fragaria ananassa* L.) leaf extract (FA–AgNPs) without a chemical catalyst to inhibit *P. aeruginosa* and *S. aureus* isolated from COVID-19 patients’ sputum. Furthermore, this work the first time introduces as a FA–AgNPs biosynthesis deadly agent against COVID-19 secondary bacterial infection stains (Scheme 1A).

## 2. Materials and methods

### 2.1. Materials

Muller-Hinton was purchased from Merck, Germany. Phosphate-buffered saline (PBS) and Hanks’ balanced salt solutions (HBSS) were purchased from Solarbio Science and Technology, China. Glutaraldehyde and [3-(4,5-dimethyl thiazol-2yl)-2,5-diphenyl tetrazolium bromide] (MTT) were purchased from Sinopharm (Beijing, China). The University of Malaya College of Medicine, Department of Pharmacy Center for Natural Product Research and Drug Discovery Department of Pharmacology Faculty of Medicine University of Malaya Kuala Lumpur Malaysia, provided the normal liver cell line WRL 68 Cell and the cancer cell line MCF-7. Cancer cells were nurtured and cultivated, and tests were performed on them at Al-Nahrain University’s Biotechnology Research Center.

### 2.2. Purification and isolation of pathogenic bacteria from COVID-19 patients

In this study, *Staphylococcus aureus* MRSA and *Pseudomonas aeruginosa* were isolated and diagnosed from hospitalized COVID-19 patients’ sputum at Salah El-Din Military Hospital, the bacteria were identified and diagnosed based on morphological characteristics and biochemical examinations according to the standard methods of diagnosis and confirmed with the Vitek 2 compact (Harrigan and McCance, 1976; Procop et al., 2020).

### 2.3. Culture conditions of the microorganism

*Staphylococcus aureus* and *P. aeruginosa* were cultured on the Muller-Hinton agar medium at 37 °C for 24 h. The colonization was compiled and assembled in HBSS (pH 6.0) to 0.06 at 600 nm (OD_600_), which corresponded to ∼10^6^ (CFU) mL^–1^ *via* UV–Vis spectroscopy (Varian Cary-100 Konc, Varian, Australia) and subsequently used in the following experiments.

### 2.4. Preparation of *Fragaria ananassa* L. leaves extract

*Fragaria ananassa* L. leaves were picked and washed four times with Milli-Q water. Then, 1 g of fresh *F.ananassa* L. leaves were mashed in 100 ml of distilled water in a 500 ml beaker, the plant extract was heated for 5 min. Finally, fresh leaves extract was filtered using a Whatman No.1 filter paper and kept in the refrigerator at 4°C (Gauthami et al., 2015).

### 2.5. Biosynthesis of FA-AgNPs

Aqueous silver nitrate (AgNO_3_) solution was prepared at a concentration of 1 mM (0.0421 g of AgNO_3_ was dissolved in 100 ml of Milli-Q water). Then, the prepared solution was kept in opaque glass bottles to ensure that the silver does not self-oxidize. Then, 1 ml of strawberry *F. ananassa* L. leaves extract was mixed with 50 ml of aqueous AgNO_3_ solution with vigorous stirring (100 rpm) at ambient room temperature around (25–27 °C) for 15 min then stored at room temperature for 8 h. The final solution of FA-AgNPs green synthesis was kept in the refrigerator at 4°C until use (Nagati et al., 2012) (Scheme 1B).

### 2.6. FA-AgNPs NPs distributions and characterizations

#### 2.6.1. Color change

The change of color reaction mixture was verified or checked through visual observation after the reduction of Ag^+^ to silver nanoparticles by *F. ananassa* L. leaves extract was measured according to Korbekandi et al. (2013).

#### 2.6.2. UV-visible

The UV-Visible spectra of samples diluted were measured on a spectrophotometer Varian Cary-100 Konc (Varian, Australia), at 230 V/50 Hz, at wavelengths of 200–800 nm (El-Belely et al., 2021).

#### 2.6.3. FTIR spectroscopy

The functional groups present in the produced samples were examined using an FT-IR spectrometer (JASCO FT-IR 4100 spectrometer, Hachioji, Tokyo, Japan). Produced samples combined with potassium bromide (KBr). High pressure was applied to a disk, and measurements were made with a resolution of 4.0 cm^–1^ at a wavelength of 400–4000 cm^–1^ (Sharaf et al., 2022a).

#### 2.6.4. Practical size (PS) and ζ-potential (ZP)

Dynamic light scattering used the formulas’ mean PS and ZP values to calculate (Malvern Instruments, UK). 3 ml of naked FA-AgNPs were diluted in deionized water, put in a cell cuvette, and scanned four times to get an average reading for size estimate. After three measurements, the mean and SD were determined.

#### 2.6.5. Surface morphology

Scanning electron microscope imaging was performed on the optimized samples using a JSM 6390^®^, manufactured by JEOL DATUM Ltd. in Japan. A 400 drop of FA-AgNPs was applied on an aluminum grid and allowed to dry for 5 min under a mercury lamp. Silver nanoparticles were investigated using TEM (transmission electron microscopy) equipment (TEM; TOPCON002B; Tokyo, Japan). Thin films of silver nanoparticles were produced on a copper grid covered with carbon by simply placing a small sample on the grid and wiping away any extra solution with blotting paper (Hou et al., 2021).

### 2.7. Inhibitory efficacy of FA-AgNPs against bacterial

The Muller–Hinton agar medium was prepared; the bacterial suspension was smeared on it with a swab, and then allowed for 5 min to dry. The well diffusion method was utilized, and three replications were made in accordance with the procedure (Dipankar and Murugan, 2012), then made 5 holes. Considering one of the pits as standard control, then 50 microliters of FA-AgNPs were added in each pit at successive concentrations (0.25, 0.50, 0.75, and 1 mM), then, placed in the incubator for 24 h at 37°C. The inhibitory efficacy of FA-AgNPs with a regular ruler, and the diameter of the inhibitory area were used to calculate the effectiveness against bacteria.

### 2.8. *In vitro* cellular biocompatibility study

MTT was used to analyze the cytotoxicity of the cancer cell MCF-7 and the normal liver cell WRL-68 lines. After incubation, the medium from the wells was removed. MTT (6 mg/mL^–1^) was then added to each well. The cells were layered into 60 μl of DMSO (dimethylsulfoxide), which dissolved when the medium was washed once more. Using a microplate reader, the specimens’ absorbance spectra were measured at a wavelength of 595 nm (Wang Y. et al., 2017).


$$\begin{array}{l} \%\text{Cell viability}=(\text{OD test}-\text{OD blank})/\\ \;(\text{OD control}-\text{OD blank}) \end{array}$$

where OD optical density, test, control, and blank refer to the wells without WRL-68 and MCF-7 cells, respectively, while test, control, and blank refer to the cells exposed to the FA-AgNPs sample (Berridge and Tan, 1993).

### 2.9. Statistical analysis

The results were statistically analyzed by applying the One Way ANOVA test (Analysis of variance), as the arithmetic averages of the inhibition areas were compared according to the concentrations used for the various substances whose biological inhibitory activity we tested for bacterial species using Duncan’s polynomial test at a *p < 0.05 (Gerber and Voelkl, 2012).

## 3. Results and discussions

### 3.1. Characterization of FA extract

Due to plant extracts’ non-toxic and safe metabolite content, they have attracted a lot of interest from different biological sources. Plants primarily use phenolic compounds, flavonoids, ketones, aldehydes, tannins, terpenoids, and organic acids as mediating chemicals to reduce silver ions. These characteristics have led to the application of FA extract in producing several nanomaterials, including gold, silver, copper, and iron nanoparticles (Bayat et al., 2021).

**FIGURE 2:**
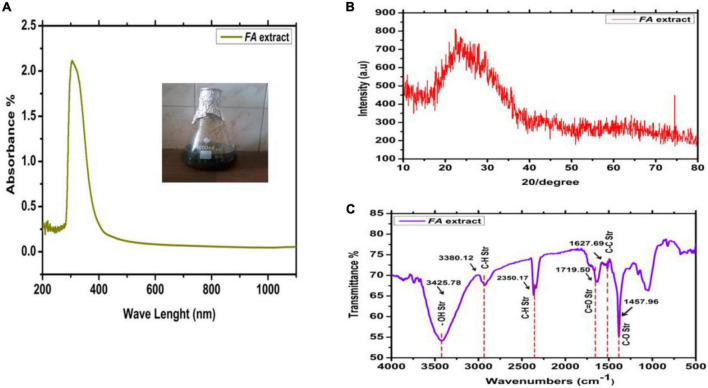
Characterization of strawberry leaf extract. **(A)** *UV-Visible*; **(B)** XRD; and **(C)** FTIR.

In the current investigation, the biological extract was made from FA leaves, and its phytochemical composition was evaluated before the AgNPs preparation (Figure 1). UV-Vis spectroscopy, FTIR, and XRD studied the Characterization of FA extract. The results are shown in Figure 2. UV-Vis spectroscopy was used to characterize the water leaves extract strawberry. Figure 2A shows the UV-visible spectrograph the spectroscopic examination proved that the highest absorption peak appeared at 378 nanometers. This spectroscopy supports the prior research by Lee et al. (2005) discovered that all extracts had a peak between 300 and 320 nm, which may have been caused by strawberry-common water-soluble phenolic or taste components extracted by pH buffers.

Furthermore, the infrared spectrum of water leaves strawberry extracted using the green synthesis method revealed a broad band at 3425 cm^–1^ belonging to the hydroxyl (OH) group, the appearance of a band at 3380 cm^–1^ belonging to the aliphatic (C-H) group, and the appearance of a band at 2350 cm^–1^ belonging to the aromatic (AR) group. A band belonging to the amide carbonyl group (*C* = O) appears at frequency 1719 cm^–1^ for the carboxylic acid carbonyl group (*C* = O), a band belonging to a group (*C* = C) appears at frequency 1627 cm^–1^, and a band belonging to a group (C-O) appears at frequency 1457 cm^–1^ (Figure 2B).

### 3.2. FA-AgNPs manufacturing strategy of preparation

**FIGURE 3:**
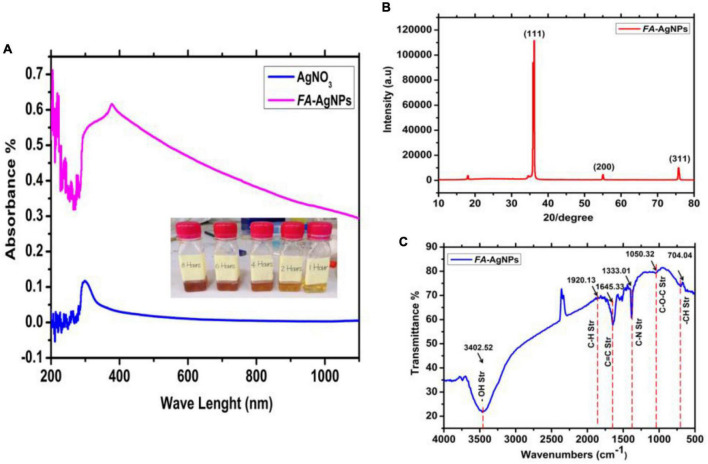
**(A)** UV-Vis spectroscopy and color change of silver nanoparticles synthesized by green synthesis method at room temperature. **(B)** XRD pattern of bare AgNPs; **(C)** FTIR.

AgNO_3_ solution mixture with FA leaves extract, the color change was monitored in glass tubes at room temperature. The increase in reaction time led to the reduction of Ag^+^ ions to silver nanoparticles. Moreover, the color of the solution was changed very clearly, which ranged from light yellow in the first hour even to dark brown in the 8 h, through a different change in color of the reduced reaction (Figure 3A). According to previous studies, the color change is the first sign of AgNO_3_ (Dipankar and Murugan, 2012; Shanmugavadivu et al., 2014). The different phytochemicals included in the leaf extract function as reducing and stabilizing agents for the production of NPs. The interaction of these phytochemicals with metal ions resulted in the production of brown color precipitates, assuring the synthesis of AgNPs (Anchan et al., 2019). Previous studies have demonstrated that phytochemicals such flavonoids and phenolic compounds directly contribute to converting Ag^+^ ions into Ag^0^ (Jain and Mehata, 2017; Devi et al., 2019). Additionally, polyhydroxy substances, particularly flavonoids, have a strong propensity to chelate metal ions by creating stable complexes through their many hydroxyl groups and the carbonyl moiety, leading to the creation of silver nanoparticles (Sahu et al., 2016; Marslin et al., 2018).

### 3.3. Characterization of FA-AgNPs extract

#### 3.3.1. UV-Vis characterization of FA-AgNPs

UV-Vis spectroscopy was used to characterize FA-AgNPs produced by aqueous extracts of blackberry and raspberry leaves. Actually, surface plasmon resonance (SPR) at around 420 nm and color variations in the reaction mixture make UV-Vis spectroscopy a highly popular technique for FA-AgNPs synthesis monitoring (Figure 3A).

#### 3.3.2. XRD

Figure 3B, represents the XRD patterns obtained of the synthesized AgNPs by green synthesis (cold process) from AgNO_3_ and FA leaves. We see intensities and peaks in the resultant patterns that are associated with cubic-crystalline AgNPs (the fitted XRD pattern was compared to the reference pattern COD Card Number [96-901-3054]. Another peak is associated with silver oxide NPs, which is (monoclinic Ag_3_O_4_–COD Card Number [96-151-0026]). This figure indicates the apparent crystal systems, angles, and Miller’s index. The exposure of the prepared solution to air for a period of time led to oxidation and thus the appearance of intensities of aforementioned peaks and with preferential directions due to the aforementioned nano silver oxide. According to a previous study (Vazquez-Muñoz et al., 2019) would be better to move the cite to the end of the sentence; the interaction of Ag atoms with oxygen atoms or radicals during their disintegration in liquid causes the creation of Ag oxide NPs. They also stated that the type of oxide formed depends on the temperature and oxygen partial pressure.

#### 3.3.3. FTIR

The dry aqueous FA and AgNPs were examined using FTIR spectra the results are shown in Figure 3C. The O-H stretching vibration is responsible for the two materials’ distinctive absorption bands in the high-frequency range of 4000 to 3000 cm^–1^. The presence of the phenolic compounds that decrease of Ag^+^ to Ag^0^. is shown by the peak of AgNPs at 3402 cm^–1^. The polyphenols are extracted from FA leaves and transformed into the silver in the solution into nano-silver particles. The band at 1920 cm^–1^ is attributed to C–H stretching of methoxy groups by anthocyanin mixture (Negahdary et al., 2015), whereas the peak at 1645 cm^–1^ is attributed to –CH and *C* = C stretching of aromatic compounds. It is worth noting that these peaks are visible in both materials (FA and AgNPs).

However, the pH was changed to a value larger than 12, making the nanoparticles’ peaks more noticeable. The anthocyanins may be chemically altered in this alkaline environment, which also aided in the synthesis of AgNPs. The amine groups C–N (Balavijayalakshmi and Ramalakshmi, 2017), and C–O–C from the synthesis using the FA and AgNO_3_ solution may be linked to the new bands at 1333 cm^–1^ and 1050 cm^–1^ on the AgNPs. This accords with (Farahani et al., 2022), and a beam from the group (–CH) also appeared at a frequency 709 cm^–1^ (Figure 3).

### 3.4. Characterization of FA-AgNPs dispersions

#### 3.4.1. Practical size (PS), PDI, and ζ-potential

**FIGURE 4:**
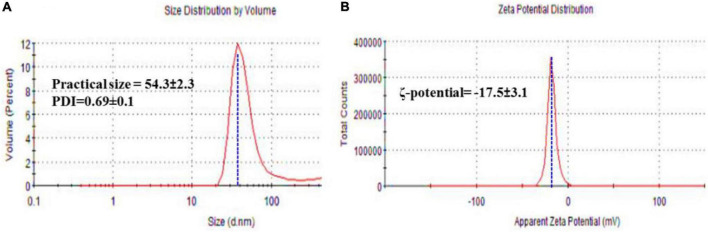
**(A)** Hydrodynamic size, and polydispersity index (PDI); and **(B)** ζ-potential of FA-AgNPs. Numerical data are reported as mean ± SD ζ-potential (*n* = 3) and particle size and PDI (*n* = 4).

Practical size, PDI, and ζ-potential of FA-AgNPs were measured by dynamic light scattering (DLS). The average values obtained across all systems revealed a PS distribution, as illustrated in Figure 4. FA-AgNPs were around ∼53 nm in size, and their PDI values were 0.69 ± 0.1, respectively (Figure 4A). Additionally, a very narrow dispersed particle has PDI values between 0.3 and 0.7, which are perfect for dispersion stability and homogeneity (Park et al., 2016). Their ζ-potential values often predict the stability of nanoparticles; in this case, FA-AgNPs defining ζ-potential values were to be −17.5 ± 3.1 mV (Figure 4B). In addition, due to electrostatic balancing, the ζ-potential value greater than −30 mV is regarded as stable. The ionization of the phenolic hydroxyl groups in the capping moieties at alkaline pH is thought to be the cause of FA-AgNPs greater negative ζ-potential charge (AbdelHamid et al., 2013). This generated a repellent barrier that prevented FA-AgNPs from aggregating and enhanced their colloidal stability.

#### 3.4.2. Transmation electron microscope (TEM)

**FIGURE 5:**
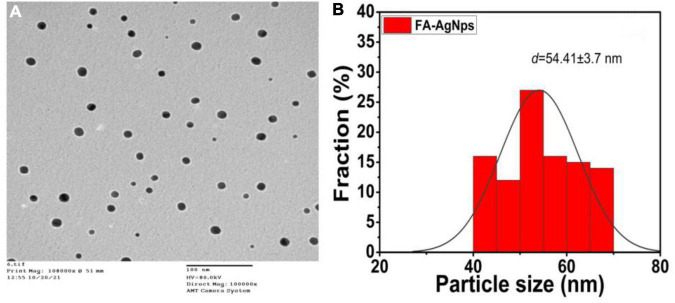
**(A)** Average particle size and size distribution of prepared samples FA-AgNPs (scale bar = 100 nm) measured by TEM; **(B)** data of size distribution is presented as means ± SD (*n* = 3).

In the current work, the topology of FA-Ag NPs was analyzed by TEM, which revealed the synthesis of monodisperse spherical NPs, as shown in Figure 5. FA flower aqueous extract was used to synthesize AgNPs, which were aggregated, spherical in form, and with an average size of between ∼45 and ∼51 nm (Figure 5A); The AgNP size was within acceptable bounds, as shown by Figure 4 of the AgNPs size distribution, which shows that the average particle size of 54.41 ± 3.7 nm (Figure 5B). The biogenic silver nanoparticles were nearly twice as large as those reported in a recent study, which found that *Phyllanthus emblica* fruit extract encouraged the creation of silver nanoparticles with an average diameter of 48.1 nm (Masum et al., 2019). Fresh *Arbutus unedo* leaf extract has been used to create green silver nanoparticles of similar sizes, with typical size diameters ranging from 40 to 58 nm (Skandalis et al., 2017). As shown in our current work, previous findings generally support the notion that using aqueous leaf extract of FA leaves to create silver nanoparticles is an efficient and environmentally benign method.

#### 3.4.3. Scanning electron microscope (SEM) and EDX analysis of FA-AgNPs

**FIGURE 6:**
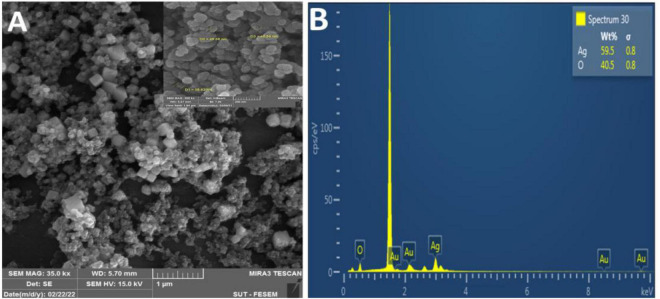
**(A)** SEM image of synthesized FA-Ag NPs (scale bar 1 μm); **(B)** EDAX spectrum for aqueous leaf extract synthesized FA-Ag NPs. The vertical axis displays the number of X-ray counts while the horizontal axis displays energy in kilo electron volts.

Scanning electron microscope pictures revealed poly aggregation nanoparticles with a range of spherical and hexagonal forms with an average size of ∼40– ∼55 nm (Figure 6A). Using energy-dispersive X-ray spectroscopy (EDX) research, the elemental mapping of the biogenic Ag NPs was discovered. According to an EDX examination, the elements oxygen (40.46%) and silver (59.54%) were present (Figure 6B). Additionally, a strong peak at 1.44 keV was seen, suggesting the presence of silver (Au), and two other peaks at 3.00 and 3.20 keV, respectively, were attributed to the presence of silver (Ag).

Our results corroborated those of Okaiyeto et al. (2019), who identified the presence of silver and the element chloride in AgNPs made from an aqueous leaf extract of *Oedera genistifolia* (Okaiyeto et al., 2019). According to a prior work (Femi-Adepoju et al., 2019), many components identified by EDX analysis, such as Si, Au, and Cl, were found to function as capping agents of the biogenic AgNPs collectively.

### 3.5. Screening antibacterial efficiency of FA-AgNPs

**FIGURE 7:**
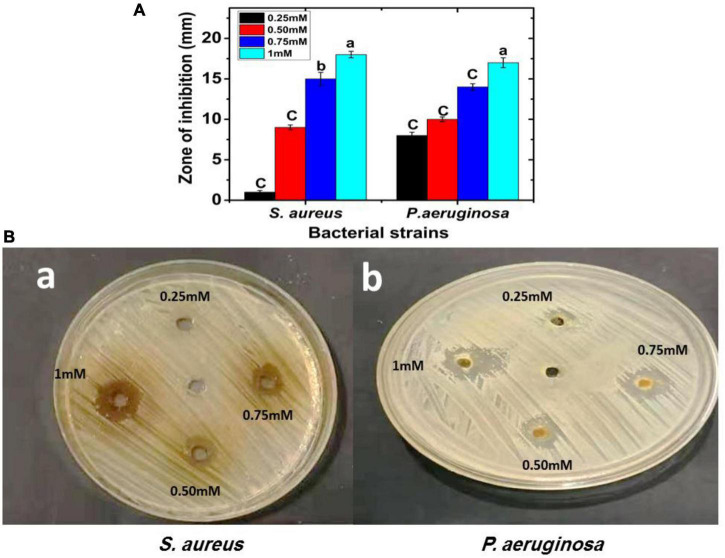
FA-AgNPs inhibit *P. aeruginosa* and *S. aureus* bacterial growth; the inhibitory effect of FA-AgNPs compared to control were determined *in vitro* by agar well diffusion assay; **(A)** zone of inhibition (mm) carve. Values in each column followed by different letters are significantly different^a,b^ (*P* < 0.05). Statistical differences between samples in Duncan test; **(Ba,b)** *S. aureus and P. aeruginosa* bacterial zone of inhibition (mm) plate with different concentrations 0.25, 0.50, 0.75, and 1 mM of FA-AgNPs.

Using a disk diffusion experiment, FA-AgNPs suspensions of different concentrations were examined for antibacterial activity against *P. aeruginosa* and *S. aureus* grown on Muller-Hinton agar medium at 37°C for 24 h. Figure 6 illustrates how the antibacterial agent (NPs) may break apart bacterial cells. At doses of 75 and 100 (mM/disk), the MRSA strain had the greatest sensitivity to FA-AgNPs, recording inhibitory zone diameters of 15.08 and 18.03 mm, respectively (Figures 7A, Ba).

These findings were strikingly similar to those reported by Yassin et al. (2022), who found that *Origanum majorana* seed extract-derived AgNPs were antibacterial effective against MRSA strains at concentrations of 75 and 100 (mM/disk), with inhibition zones measuring 19.68 and 17.12 mm, respectively. Additionally, at doses of 75 and 100 (mM/disk), the *P. aeruginosa* strain showed remarkable sensitivity to the biosynthesized AgNPs, recording a suppressive zone width of 14.68 and 17.15 mm, respectively (Figures 7A, Bb).

These results corroborated Khalil et al. (2021), who revealed the antibacterial efficacy of green AgNPs produced from extracts of ginger, onion, and sidr and recorded suppressive exhibited varied inhibitory zone sizes ranging from 11 to 17 mm. These outcomes could result from silver nanoparticles’ ability to continuously release silver ions, which might function as a microbe-killing mechanism (Qamer et al., 2021; Wright et al., 2022). Furthermore, due to electrostatic attraction and affinity for sulfur proteins, silver ions can adhere to the cytoplasmic membrane and cell wall (Tang and Zheng, 2018). The cytoplasmic membrane’s permeability may increase due to the connected ions, disrupting the bacterial envelope (Santos et al., 2018). Once free silver ions are ingested by cells, respiratory enzymes may become inactive, leading to reactive oxygen species without adenosine triphosphate production (Hamad et al., 2020).

Reactive oxygen species can also be aided in triggering cell membrane rupture and DNA modification (Singh et al., 2018). The interaction of silver ions with sulfur and phosphorus, two essential DNA building blocks, results in issues with DNA replication, cell growth, and, ultimately, microbial cell death (Makvandi et al., 2020). Silver ions can also stop the synthesis of proteins by denaturing ribosomes in the cytoplasm (Sharma et al., 2021). Due to their increased surface area, smaller AgNPs, like those reported in the current study and having a spherical form, are more likely to release silver. AgNPs’ nanoscale size also allows them to pass through bacterial cell walls and alter the structure of the cell membrane. Organelle rupture and potential cell lysis can result from the cytoplasmic membrane becoming denatured (Uddin et al., 2022).

### 3.6. Screening cytotoxicity study and anticancer of FA-AgNPs

**FIGURE 8:**
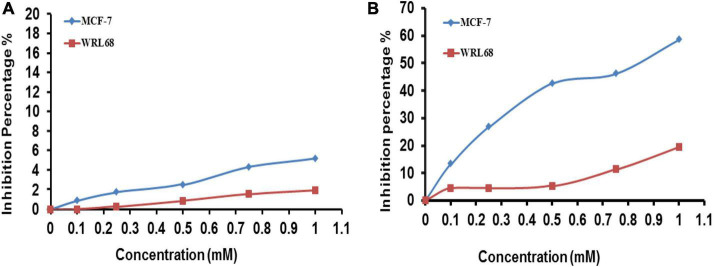
Viability of WRL68 and MCF-7 breast cancer cell lines, cells treated with different concentrations of panel **(A)** *F. ananassa* extract; **(B)** FA-AgNPs. Numerical data are reported as the mean ± SD (*n* = 3).

Concerns over the biological effects of AgNPs extensive use as well as potential hazards to the environment and public health, are on the rise. Because of their small size, nanoparticles may readily infiltrate live beings’ cells, which can result in a number of cell damages (Greulich et al., 2011; Liz et al., 2015). Nanoparticles have been extensively explored as a result of growing worries about their potential cytotoxicity and genotoxicity (Chairuangkitti et al., 2013; Składanowski et al., 2016). Both the normal cell line (WRL-68) and the cancer cell line (MCF-7) were treated for 24 h at 37°C with doses ranging from 0.1 to 1 mM) of FA extract (Figure 8A) and FA-AgNPs (Figure 8B), along with the control sample without treatment for comparison. The magnitude of the toxicological impact was also assessed by calculating the percentage of growth that was inhibited compared to the control (100% growth).

The 3T3 Phototox software was used to process the absorbance values obtained following the capture of the red dye and the corresponding doses of FA-AgNPs employed in the cytotoxicity studies to calculate the IC50 of produced samples in various cell lines. At the highest quantities of the extract evaluated in this study, very little toxicity to the normal cell lines WRL-68 and MCF-7 was seen (Figure 8A). Additionally, FA-AgNPs demonstrated dose-dependently enhanced inhibition rate and reduced cell viability on WRL-68 cells at a high dose of <1 mM and on MCF-7 cells at a low dose of >1 mM (IC_50_, around 0.9 mM). This data can suggest a certain level of safety for greater FA-AgNPs concentrations in coating medical equipment, as the concentration of 1 mM, where silver ions should be released slowly, is probably never achieved (Figure 8B). These findings were very comparable to those of Balan et al. (2021).

There are some limitations to the current investigation. Therefore, should be investigated further in this work, first, *in vitro* impact of specified formulations on pre-established bacteria biofilms. Second, future research, in conjunction with FA-AgNPs, will give the knowledge required to forecast synergism and disruptive effects on an established biofilm-based infection. Furthermore, we recommend doing *in vivo* experiments to establish the effectiveness of our developed formulations along with cytotoxicity.

## 4. Conclusion

In order to increase AgNPs efficiency, the co-sedimentation method was used to prepare silver nanoparticles from *F. ananassa* L. leaves extract (FA-AgNPs). FA-AgNPs UV-Visible showed surface plasmon resonance at 420 nm. XRD and FTIR results showed intensities and peaks in the resultant patterns that are associated with cubic-crystalline AgNPs. The zeta size of FA-AgNPs was around ∼53 nm and negative ζ-potential charge. Furthermore, the surface appearance by TEM, SEM, and EDAX spectrum depicted that the formed of FA-AgNPs exhibited good homogeneity and spherical size. FA-AgNP was employed with the MCF-7 breast tumor cell line, doses demonstrated considerable cytotoxic effects, decreased cell viability, and increased inhibition rate in a dose-dependent manner on MCF-7 cells at >1 mM (IC50, approximately 0.9 mM), as well as antibacterial efficacy against the chosen Gram-negative and Gram-positive pathogens. In order to combat the multi-drug-resistant bacterial strains that were discovered in the sputum of COVID-19 patients, FA-AgNPs may be a useful option to develop. Applications of FA-AgNPs may result in important discoveries in various domains, including antimicrobial systems and medical equipment used in institutions authorized to isolate COVID-19 patients.

## Data availability statement

The original contributions presented in this study are included in the article/supplementary material, further inquiries can be directed to the corresponding authors.

## Author contributions

NH, AH, SR, FA, MS, and SZ: conceptualization. NH, AH, SR, FA, and MS: methodology and investigation. NH, AH, SR, FA, MS, SZ, SY, NE, and AE: software. SZ, SY, AMA, AA-E, AA, NE, and AE: validation. NH, AH, SR, FA, MS, SZ, and SY: formal analysis. NH, AH, SR, FA, MS, SZ, SY, AMA, AA-E, AA, NE, and AE: data curation, writing – original draft preparation, and supervision. NH, AH, SR, FA, MS, and AE: writing – review and editing. FA, MS, and AE: project administration. NH, FA, MS, NE, and AE: funding acquisition. All authors read and agreed to the published version of the manuscript.

## References

[B1] AbdelHamid A. Al-Ghobashy M. Fawzy M. Mohamed M. Abdel-Mottaleb M. (2013). Phytosynthesis of Au, Ag, and Au–Ag bimetallic nanoparticles using aqueous extract of sago pondweed (*Potamogeton pectinatus* L.). *ACS Sustain. Chem. Eng.* 1 1520–1529. 10.1021/sc4000972

[B2] Abdoli A. Falahi S. Kenarkoohi A. (2021). COVID-19-associated opportunistic infections: A snapshot on the current reports. *Clin. Exp. Med.* 22 327–346. 10.1007/s10238-021-00751-7 34424451 PMC8381864

[B3] Alshawwa S. Mohammed E. Hashim N. Sharaf M. Selim S. Alhuthali H. (2022). In Situ biosynthesis of reduced alpha hematite (α-Fe2O3) nanoparticles by *Stevia Rebaudiana* L. leaf extract: Insights into antioxidant, antimicrobial, and anticancer properties. *Antibiotics* 11:1252. 10.3390/antibiotics11091252 36140030 PMC9495369

[B4] Anchan S. Pai S. Sridevi H. Varadavenkatesan T. Vinayagam R. Selvaraj R. (2019). Biogenic synthesis of ferric oxide nanoparticles using the leaf extract of *Peltophorum pterocarpum* and their catalytic dye degradation potential. *Biocatal. Agric. Biotechnol.* 20:101251. 10.1016/j.bcab.2019.101251

[B5] Awad M. de Jager A. (2003). Influences of air and controlled atmosphere storage on the concentration of potentially healthful phenolics in apples and other fruits. *Postharvest Biol. Technol.* 27 53–58. 10.1016/S0925-5214(02)00189-8

[B6] Balan L. Chandrasekaran S. Gajendiran M. Nanjian R. (2021). Synthesis of silver nanoparticles from *Pedalium murex* L. and its antiproliferative activity against breast cancer (MCF-7) cells. *J. Mol. Struct.* 1242:130695. 10.1016/j.molstruc.2021.130695

[B7] Balavijayalakshmi J. Ramalakshmi V. (2017). *Carica papaya* peel mediated synthesis of silver nanoparticles and its antibacterial activity against human pathogens. *J. Appl. Res. Technol.* 15 413–422. 10.1016/j.jart.2017.03.010

[B8] Bapat R. Chaubal T. Joshi C. Bapat P. Choudhury H. Pandey M. (2018). An overview of application of silver nanoparticles for biomaterials in dentistry. *Mater. Sci. Eng. C Mater. Biol. Appl.* 91 881–898. 10.1016/j.msec.2018.05.069 30033323

[B9] Bassetti M. Magnasco L. Vena A. Portunato F. Giacobbe D. (2022). Methicillin-resistant *Staphylococcus aureus* lung infection in coronavirus disease 2019: How common? *Curr. Opin. Infect. Dis.* 35 149–162. 10.1097/QCO.0000000000000813 35125396 PMC8900893

[B10] Bayat M. Zargar M. Astarkhanova T. Pakina E. Ladan S. Lyashko M. (2021). Facile biogenic synthesis and characterization of seven metal-based nanoparticles conjugated with phytochemical bioactives using *Fragaria ananassa* leaf extract. *Molecules* 26:3025. 10.3390/molecules26103025 34069463 PMC8159137

[B11] Berridge M. Tan A. (1993). Characterization of the cellular reduction of 3-(4, 5-dimethylthiazol-2-yl)-2, 5-diphenyltetrazolium bromide (MTT): Subcellular localization, substrate dependence, and involvement of mitochondrial electron transport in MTT reduction. *Arch. Biochem. Biophys.* 303 474–482. 10.1006/abbi.1993.1311 8390225

[B12] Bosch A. Biesbroek G. Trzcinski K. Sanders E. Bogaert D. (2013). Viral and bacterial interactions in the upper respiratory tract. *PLoS Pathog.* 9:e1003057. 10.1371/journal.ppat.1003057 23326226 PMC3542149

[B13] Chairuangkitti P. Lawanprasert S. Roytrakul S. Aueviriyavit S. Phummiratch D. Kulthong K. (2013). Silver nanoparticles induce toxicity in A549 cells via ROS-dependent and ROS-independent pathways. *Toxicol. Vitro* 27 330–338. 10.1016/j.tiv.2012.08.021 22940466

[B14] Chong W. Saha B. Ramani A. Chopra A. (2021). State-of-the-art review of secondary pulmonary infections in patients with COVID-19 pneumonia. *Infection* 49 591–605. 10.1007/s15010-021-01602-z 33709380 PMC7951131

[B15] Darroudi M. Hakimi M. Goodarzi E. Oskuee R. (2014). Superparamagnetic iron oxide nanoparticles (SPIONs): Green preparation, characterization and their cytotoxicity effects. *Ceram. Int.* 40 14641–14645. 10.1016/j.ceramint.2014.06.051

[B16] Devi H. Boda M. Shah M. Parveen S. Wani A. (2019). Green synthesis of iron oxide nanoparticles using *Platanus orientalis* leaf extract for antifungal activity. *Green Process. Synth.* 8 38–45. 10.1515/gps-2017-0145

[B17] Dipankar C. Murugan S. (2012). The green synthesis, characterization and evaluation of the biological activities of silver nanoparticles synthesized from *Iresine herbstii* leaf aqueous extracts. *Colloids Surf. B* 98 112–119. 10.1016/j.colsurfb.2012.04.006 22705935

[B18] Duployez C. Le Guern R. Tinez C. Lejeune A. Robriquet L. Six S. (2020). Panton-valentine leukocidin–secreting *Staphylococcus aureus* pneumonia complicating COVID-19. *Emerg. Infect. Dis.* 26:1939. 10.3201/eid2608.201413 32298228 PMC7392470

[B19] El-Belely E. Farag M. Said H. Amin A. Azab E. Gobouri A. (2021). Green synthesis of zinc oxide nanoparticles (ZnO-NPs) using *Arthrospira platensis* (Class: *Cyanophyceae*) and evaluation of their biomedical activities. *Nanomaterials* 11:95. 10.3390/nano11010095 33406606 PMC7823323

[B20] El-Mesallamy A. Hussein S. Gerby M. Abd El Azim M. (2013). Phenolic composition and biological activities of methanolic extract of strawberry leaves (*Fragaria ananassa*). *Nat. Prod.* 9 251–265.

[B21] Farahani A. Hamdi S. Mirzaee A. (2022). GC/MS analysis and phyto-synthesis of silver nanoparticles using *Amygdalus spinosissima* extract: Antibacterial, antioxidant effects, anticancer and apoptotic effects. *Avicenna J. Med. Biotechnol.* 14 223–232. 10.18502/ajmb.v14i3.9829 36061132 PMC9376992

[B22] Femi-Adepoju A. Dada A. Otun K. Adepoju A. Fatoba O. (2019). Green synthesis of silver nanoparticles using terrestrial fern (*Gleichenia Pectinata* (Willd.) C. Presl.): Characterization and antimicrobial studies. *Heliyon* 5:e01543. 10.1016/j.heliyon.2019.e01543 31049445 PMC6479216

[B23] Gauthami M. Srinivasan N. Goud N. Boopalan K. Thirumurugan K. (2015). Synthesis of silver nanoparticles using *Cinnamomum zeylanicum* bark extract and its antioxidant activity. *Nanosci. Nanotechnol. Asia* 5 2–7. 10.2174/221068120501150728103209

[B24] Gellatly S. Hancock R. (2013). *Pseudomonas aeruginosa*: New insights into pathogenesis and host defenses. *Pathog. Dis.* 67 159–173. 10.1111/2049-632X.12033 23620179

[B25] Gerber S. Voelkl K. (2012). *The SPSS guide to the new statistical analysis of data.* New York, NY: Springer Science & Business Media.

[B26] Greulich C. Diendorf J. Simon T. Eggeler G. Epple M. Köller M. (2011). Uptake and intracellular distribution of silver nanoparticles in human mesenchymal stem cells. *Acta Biomater.* 7 347–354. 10.1016/j.actbio.2010.08.003 20709196

[B27] Hamad A. Khashan K. Hadi A. (2020). Silver nanoparticles and silver ions as potential antibacterial agents. *J. Inorg. Organomet. Polym. Mater.* 30 4811–4828.

[B28] Hanada S. Pirzadeh M. Carver K. Deng J. (2018). Respiratory viral infection-induced microbiome alterations and secondary bacterial pneumonia. *Front. Immunol.* 9:2640. 10.3389/fimmu.2018.02640 30505304 PMC6250824

[B29] Harrigan W. McCance M. (1976). *Laboratory methods in food and dairy microbiology.* London: Academic Press Inc. Ltd.

[B30] He F. Xia X. Nie D. Yang H. Jiang Y. Huo X. (2020). Respiratory bacterial pathogen spectrum among COVID-19 infected and non–COVID-19 virus infected pneumonia patients. *Diagn. Microbiol. Infect. Dis.* 98:115199. 10.1016/j.diagmicrobio.2020.115199 32979617 PMC7470696

[B31] Hou Y. Kovács N. Xu H. Sun C. Erni R. de Jesús Gálvez-Vázquez M. (2021). Limitations of identical location SEM as a method of degradation studies on surfactant capped nanoparticle electrocatalysts. *J. Catal.* 394 58–66. 10.1016/j.jcat.2020.12.006

[B32] Jain S. Mehata M. (2017). Medicinal plant leaf extract and pure flavonoid mediated green synthesis of silver nanoparticles and their enhanced antibacterial property. *Sci. Rep.* 7:15867. 10.1038/s41598-017-15724-8 29158537 PMC5696514

[B33] Jakobek L. Seruga M. Novak I. Medvidovic-Kosanovic M. (2007). Flavonols, phenolic acids and antioxidant activity of some red fruits. *Dtsch. Lebensm. Rundsch.* 103 369–377.

[B34] Jose M. Desai K. (2020). Fatal superimposed bacterial sepsis in a healthy coronavirus (COVID-19) patient. *Cureus* 12:e8350. 10.7759/cureus.8350 32617223 PMC7325395

[B35] Kandeel M. Sharaf M. Hamad A. Babalghith A. Abdalla M. Arif M. (2022). Novel copper oxide bio-nanocrystals to target outer membrane lectin of vancomycin-resistant *Enterococcus faecium* (VREfm): In silico, bioavailability, antimicrobial, and anticancer potential. *Molecules* 27:7957. 10.3390/molecules27227957 36432057 PMC9696412

[B36] Kaul D. Rathnasinghe R. Ferres M. Tan G. Barrera A. Pickett B. (2020). Microbiome disturbance and resilience dynamics of the upper respiratory tract during influenza A virus infection. *Nat. Commun.* 11:3132. 10.1038/s41467-020-16429-9 32546784 PMC7298031

[B37] Kesharwani P. Gorain B. Low S. Tan S. Ling E. Lim Y. (2018). Nanotechnology based approaches for anti-diabetic drugs delivery. *Diabetes Res. Clin. Pract.* 136 52–77. 10.1016/j.diabres.2017.11.018 29196152

[B38] Khalil M. El Maghraby G. Sonbol F. Allam N. Ateya P. Ali S. (2021). Enhanced efficacy of some antibiotics in presence of silver nanoparticles against multidrug resistant *Pseudomonas aeruginosa* recovered from burn wound infections. *Front. Microbiol.* 12:648560. 10.3389/fmicb.2021.648560 34616370 PMC8488261

[B39] Khan I. Tabassum S. Ikram M. Zia M. (2018). Antioxidant, cytotoxicity, protein kinase inhibition and antibacterial activities of *Fragaria*× *ananassa* leaves. *Pak. J. Pharm. Sci.* 31 1423–1429. 30033429

[B40] Khorrami S. Zarrabi A. Khaleghi M. Danaei M. Mozafari M. (2018). Selective cytotoxicity of green synthesized silver nanoparticles against the MCF-7 tumor cell line and their enhanced antioxidant and antimicrobial properties. *Int. J. Nanomed.* 13:8013. 10.2147/IJN.S189295 30568442 PMC6267361

[B41] Korbekandi H. Ashari Z. Iravani S. Abbasi S. (2013). Optimization of biological synthesis of silver nanoparticles using *Fusarium oxysporum*. *Iran. J. Pharm. Res.* 12:289. 24250635 PMC3813263

[B42] Lansbury L. Lim B. Baskaran V. Lim W. (2020). Co-infections in people with COVID-19: A systematic review and meta-analysis. *J. Infect.* 81 266–275. 10.1016/j.jinf.2020.05.046 32473235 PMC7255350

[B43] Lee J. Durst R. Wrolstad R. Kupina C. Jd S. (2005). Determination of total monomeric anthocyanin pigment content of fruit juices, beverages, natural colorants, and wines by the pH differential method: Collaborative study. *J. AOAC Int.* 88 1269–1278. 10.1093/jaoac/88.5.1269 16385975

[B44] Liston A. Cronn R. Ashman T. (2014). *Fragaria*: A genus with deep historical roots and ripe for evolutionary and ecological insights. *Am. J. Bot.* 101 1686–1699. 10.3732/ajb.1400140 25326614

[B45] Liz R. Simard J. Leonardi L. Girard D. (2015). Silver nanoparticles rapidly induce atypical human neutrophil cell death by a process involving inflammatory caspases and reactive oxygen species and induce neutrophil extracellular traps release upon cell adhesion. *Int. Immunopharmacol.* 28 616–625. 10.1016/j.intimp.2015.06.030 26241783

[B46] Luyt C. Sahnoun T. Gautier M. Vidal P. Burrel S. Pineton de Chambrun M. (2020). Ventilator-associated pneumonia in patients with SARS-CoV-2-associated acute respiratory distress syndrome requiring ECMO: A retrospective cohort study. *Ann. Intensive Care* 10:158. 10.1186/s13613-020-00775-4 33230710 PMC7682692

[B47] Makvandi P. Wang C. Zare E. Borzacchiello A. Niu L. Tay F. (2020). Metal-based nanomaterials in biomedical applications: Antimicrobial activity and cytotoxicity aspects. *Adv. Funct. Mat.* 30:1910021. 10.1002/adfm.201910021

[B48] Marslin G. Siram K. Maqbool Q. Selvakesavan R. Kruszka D. Kachlicki P. (2018). Secondary metabolites in the green synthesis of metallic nanoparticles. *Materials* 11:940. 10.3390/ma11060940 29865278 PMC6024997

[B49] Masum M. Islam M. Siddiqa M. Ali K. Zhang Y. Abdallah Y. (2019). Biogenic synthesis of silver nanoparticles using *Phyllanthus emblica* fruit extract and its inhibitory action against the pathogen *Acidovorax oryzae* strain RS-2 of rice bacterial brown stripe. *Front. Microbiol.* 10:820. 10.3389/fmicb.2019.00820 31110495 PMC6501729

[B50] Monegro A. Muppidi V. Regunath H. (2021). *Hospital acquired infections.* Treasure Island, FL: StatPearls Publishing Copyright. 28722887

[B51] Mulcahy M. McLoughlin R. (2016). *Staphylococcus aureus* and influenza a virus: Partners in coinfection. *Mbio* 7 e2068–e2016. 10.1128/mBio.02068-16 27965455 PMC5156308

[B52] Nagati V. Alwala J. Koyyati R. Donda M. Banala R. Padigya P. (2012). Green synthesis of plant-mediated silver nanoparticles using *Withania somnifera* leaf extract and evaluation of their antimicrobial activity. *Asian Pac. J. Trop. Biomed.* 2 1–5.23569824

[B53] Negahdary M. Omidi S. Eghbali-Zarch A. Mousavi S. Mohseni G. Moradpour Y. (2015). Plant synthesis of silver nanoparticles using *Matricaria chamomilla* plant and evaluation of its antibacterial and antifungal effects. *Biomed. Res.* 26 764–799.

[B54] Nishtha P. Richa P. Rao A. (2017). Phytochemical analysis and anatomical study of two species of *Cestrum* from chandigarh. *Int. J. Pharm. Sci. Res.* 8 5234–5240.

[B55] Okaiyeto K. Ojemaye M. Hoppe H. Mabinya L. Okoh A. (2019). Phytofabrication of silver/silver chloride nanoparticles using aqueous leaf extract of *Oedera genistifolia*: Characterization and antibacterial potential. *Molecules* 24:4382. 10.3390/molecules24234382 31801244 PMC6930575

[B56] Park J. Cha S. Cho S. Park Y. (2016). Green synthesis of gold and silver nanoparticles using gallic acid: Catalytic activity and conversion yield toward the 4-nitrophenol reduction reaction. *J. Nanopart. Res.* 18:166. 10.1007/s11051-016-3466-2

[B57] Procop G. Church D. Hall G. Janda W. (2020). *Koneman’s color atlas and textbook of diagnostic microbiology.* Burlington, MA: Jones & Bartlett Publishers.

[B58] Punjabi C. Madaline T. Gendlina I. Chen V. Nori P. Pirofski L. (2021). Prevalence of methicillin-resistant *Staphylococcus aureus* (MRSA) in respiratory cultures and diagnostic performance of the MRSA nasal polymerase chain reaction (PCR) in patients hospitalized with coronavirus disease 2019 (COVID-19) pneumonia. *Infect. Control Hosp. Epidemiol.* 42 1156–1158. 10.1017/ice.2020.440 32843125 PMC7588710

[B59] Punjabi Y. Khilnani V. Damle P. (2015). The investigation of antibacterial activity of *Cestrum nocturnum*. *Pharmacophore* 6 81–87.

[B60] Qamer S. Romli M. Che-Hamzah F. Misni N. Joseph N. Al-Haj N. (2021). Systematic review on biosynthesis of silver nanoparticles and antibacterial activities: Application and theoretical perspectives. *Molecules* 26:5057. 10.3390/molecules26165057 34443644 PMC8398138

[B61] Qu J. Cai Z. Duan X. Zhang H. Cheng H. Han S. (2022). *Pseudomonas aeruginosa* modulates alginate biosynthesis and type VI secretion system in two critically ill COVID-19 patients. *Cell Biosci.* 12 1–18. 10.1186/s13578-022-00748-z 35139898 PMC8827185

[B62] Qu J. Cai Z. Liu Y. Duan X. Han S. Liu J. (2021). Persistent bacterial coinfection of a COVID-19 patient caused by a genetically adapted *Pseudomonas aeruginosa* chronic colonizer. *Front. Cell. Infect. Microbiol.* 11:641920. 10.3389/fcimb.2021.641920 33816347 PMC8010185

[B63] Ramkumar V. Pugazhendhi A. Gopalakrishnan K. Sivagurunathan P. Saratale G. Dung T. (2017). Biofabrication and characterization of silver nanoparticles using aqueous extract of seaweed enteromorpha compressa and its biomedical properties. *Biotechnol. Rep.* 14 1–7. 10.1016/j.btre.2017.02.001 28459002 PMC5397105

[B64] Rhoades N. Pinski A. Monsibais A. Jankeel A. Doratt B. Cinco I. (2021). Acute SARS-CoV-2 infection is associated with an increased abundance of bacterial pathogens, including *Pseudomonas aeruginosa* in the nose. *Cell Rep.* 36:109637. 10.1016/j.celrep.2021.109637 34433082 PMC8361213

[B65] Sahu N. Soni D. Chandrashekhar B. Satpute D. Saravanadevi S. Sarangi B. (2016). Synthesis of silver nanoparticles using flavonoids: Hesperidin, naringin and diosmin, and their antibacterial effects and cytotoxicity. *Int. Nano Lett.* 6 173–181. 10.1007/s40089-016-0184-9

[B66] Santos R. Figueiredo C. Azevedo N. Braeckmans K. De Smedt S. (2018). Nanomaterials and molecular transporters to overcome the bacterial envelope barrier: Towards advanced delivery of antibiotics. *Adv. Drug Deliv. Rev.* 136 28–48. 10.1016/j.addr.2017.12.010 29248479

[B67] Saravanan M. Barik S. MubarakAli D. Prakash P. Pugazhendhi A. (2018). Synthesis of silver nanoparticles from *Bacillus brevis* (NCIM 2533) and their antibacterial activity against pathogenic bacteria. *Microb. Pathog.* 116 221–226. 10.1016/j.micpath.2018.01.038 29407231

[B68] Seeram N. Lee R. Scheuller H. Heber D. (2006). Identification of phenolic compounds in strawberries by liquid chromatography electrospray ionization mass spectroscopy. *Food Chem.* 97 1–11. 10.1016/j.foodchem.2005.02.047

[B69] Selim S. Faried O. Almuhayawi M. Saleh F. Sharaf M. El Nahhas N. (2022). Incidence of vancomycin-resistant *Staphylococcus aureus* strains among patients with urinary tract infections. *Antibiotics* 11:408. 10.3390/antibiotics11030408 35326871 PMC8944512

[B70] Shanmugavadivu M. Kuppusamy S. Ranjithkumar R. (2014). Synthesis of pomegranate peel extract mediated silver nanoparticles and its antibacterial activity. *Am. J. Adv. Drug Deliv.* 2 174–182.

[B71] Sharaf M. Sewid A. Hamouda H. Elharrif M. El-Demerdash A. Alharthi A. (2022b). Rhamnolipid-coated iron oxide nanoparticles as a novel multitarget candidate against major foodborne e. coli serotypes and methicillin-resistant s. *aureus*. *Microbiol. Spect.* 10:e0025022. 10.1128/spectrum.00250-22 35852338 PMC9430161

[B72] Sharaf M. Arif M. Hamouda H. Khan S. Abdalla M. Shabana S. (2022a). Preparation, urease inhibition mechanisms, and anti-Helicobacter pylori activities of hesperetin-7-rhamnoglucoside. *Curr. Res. Microb. Sci.* 3:100103. 10.1016/j.crmicr.2021.100103 35024644 PMC8732090

[B73] Sharifipour E. Shams S. Esmkhani M. Khodadadi J. Fotouhi-Ardakani R. Koohpaei A. (2020). Evaluation of bacterial co-infections of the respiratory tract in COVID-19 patients admitted to ICU. *BMC Infect. Dis.* 20:646. 10.1186/s12879-020-05374-z 32873235 PMC7461753

[B74] Sharma D. Gulati S. Sharma N. Chaudhary A. (2021). Sustainable synthesis of silver nanoparticles using various biological sources and waste materials: A review. *Emerg. Mater.* 5 1649–1678. 10.1007/s42247-021-00292-5

[B75] Singh P. Garg A. Pandit S. Mokkapati V. Mijakovic I. (2018). Antimicrobial effects of biogenic nanoparticles. *Nanomaterials* 8:1009. 10.3390/nano8121009 30563095 PMC6315689

[B76] Skandalis N. Dimopoulou A. Georgopoulou A. Gallios N. Papadopoulos D. Tsipas D. (2017). The effect of silver nanoparticles size, produced using plant extract from *Arbutus unedo*, on their antibacterial efficacy. *Nanomaterials* 7:178. 10.3390/nano7070178 28698511 PMC5535244

[B77] Składanowski M. Golinska P. Rudnicka K. Dahm H. Rai M. (2016). Evaluation of cytotoxicity, immune compatibility and antibacterial activity of biogenic silver nanoparticles. *Med. Microbiol. Immunol.* 205 603–613. 10.1007/s00430-016-0477-7 27620485 PMC5093183

[B78] Sposito B. Broggi A. Pandolfi L. Crotta S. Clementi N. Ferrarese R. (2021). The interferon landscape along the respiratory tract impacts the severity of COVID-19. *Cell* 184 4953–4968.e16. 10.1016/j.cell.2021.08.016 34492226 PMC8373821

[B79] Sumaira S. Muhammad R. Rahmat A. (2011). Phenolic compounds and antioxidant activities of *Rumex hastatus* D. Don. Leaves. *J. Med. Plants Res.* 5 2755–2765.

[B80] Tang S. Zheng J. (2018). Antibacterial activity of silver nanoparticles: Structural effects. *Adv. Healthcare Mater.* 7:1701503. 10.1002/adhm.201701503 29808627

[B81] Uddin I. Parimi D. Bollu T. Bhatt C. Suresh A. (2022). “Silver Nanoparticles as Potent Multidrug-Resistant Incorporants in Biomedicine,” in *Emerging modalities in mitigation of antimicrobial resistance*, eds Akhtar N. Singh K. S. Prerna G. (Cham: Springer), 475–488. 10.1007/978-3-030-84126-3_21

[B82] Vazquez-Muñoz R. Meza-Villezcas A. Fournier P. Soria-Castro E. Juarez-Moreno K. Gallego-Hernández A. (2019). Enhancement of antibiotics antimicrobial activity due to the silver nanoparticles impact on the cell membrane. *PLoS One* 14:e0224904. 10.1371/journal.pone.0224904 31703098 PMC6839893

[B83] Wang K. Chen Y. Salido M. Kohli G. Kong J. Liang H. (2017). The rapid in vivo evolution of *Pseudomonas aeruginosa* in ventilator-associated pneumonia patients leads to attenuated virulence. *Open Biol.* 7:170029. 10.1098/rsob.170029 28878043 PMC5627047

[B84] Wang Y. Wu Y. Quadri F. Prox J. Guo L. (2017). Cytotoxicity of ZnO nanowire arrays on excitable cells. *Nanomaterials* 7:80. 10.3390/nano7040080 28387734 PMC5408172

[B85] Wright T. Vlok M. Shapira T. Olmstead A. Jean F. Wolf M. (2022). Photodynamic and Contact Killing Polymeric Fabric Coating for Bacteria and SARS-CoV-2. *ACS Appl. Mater. Interf.* 14 49–56. 10.1021/acsami.1c14178 34978405

[B86] Yang H. Zhan J. Fang B. Lyu J. (2015). Research on acute non-viral respiratory tract infection pathogens spectrum of four hundred influenza-like cases. *Chin. J. Prevent. Med.* 49 567–570. 26310346

[B87] Yassin M. Mostafa A. Al-Askar A. Al-Otibi F. (2022). Facile green synthesis of silver nanoparticles using aqueous leaf extract of *Origanum majorana* with potential bioactivity against multidrug resistant bacterial strains. *Crystals* 12:603. 10.3390/cryst12050603

